# The Toner Lectures

**Published:** 1873-04

**Authors:** 


The Toner Lectures.—The second of the Toner course of Lectures was delivered Friday evening, April 18th, before a large and appreciative audience at Washington, D. C., by Professor C. E. Brown Sequard, M. D. His subject was "Nervous Force: The Extent, Variety and Power of its Manifestations."

These lectures thus far have been a success, and Dr. Toner deserves much credit for establishing a fund for their support. The trustees of the fund are: The Secretary of the Smithsonian Institution, The Surgeon General U. S. A., The Surgeon General U. S. N., The President D. C. Medical Society and Dr. Toner.

